# Paediatric airway foreign-body removal equipment availability in sub-Saharan Africa

**DOI:** 10.4102/jcmsa.v2i1.45

**Published:** 2024-05-24

**Authors:** Fiona Kabagenyi, Alexander D. Cherches, Nina R. Patel, Samuel N. Okerosi, Mary Jue Xu, Wale L. Gellaw, Tagwa H.A. Abdalla, Felicia Tshite, Buhlebenkosi J. Hlomani, Titus Dzongodza, Reuel K. Maina, Shazia Peer, Joshua Wiedermann, Douglas R. Sidell, Taseer Din

**Affiliations:** 1 Department of Ear Nose and Throat, Faculty of Medicine, Makerere University, Kampala, Uganda; 2 Faculty of Medicine, Duke University, School of Medicine, Durham, North Carolina, United States of America; 3 Faculty of Medicine, Loyola Chicago Stritch, School of Medicine, Chicago, IL, United States of America; 4 Department of Ear, Nose and Throat, Faculty of Medicine, Kenyatta National Hospital, Nairobi, Kenya; 5 Department of Otolaryngology, Head and Neck Surgery, Faculty of Medicine, University of California, San Francisco, United States of America; 6 National Clinician Scholars Program, University of California, San Francisco, United States of America; 7 Department of Ear, Nose and Throat, Faculty of Surgery, St Paul Hospital, Addis Ababa, Ethiopia; 8 Department of Ear, Nose and Throat, Faculty of Medicine, University of Khartoum, Khartoum, Sudan; 9 Department of Ear, Nose and Throat, Faculty of Surgery, University of Pretoria, Pretoria, South Africa; 10 Department of Ear, Nose and Throat, Faculty of Surgery, Ondangwa Private, Ondangwa, Namibia; 11 Department of Ear, Nose and Throat, Faculty of Surgery, Sally Mugabe Children’s Hospital, Harare, Zimbabwe; 12 Department of Ear, Nose and Throat, Faculty of Surgery, Kenyatta National Hospital, Nairobi, Kenya; 13 Department of Surgery, Faculty of Health Sciences, University of Cape Town, Cape Town, South Africa; 14 Department of Surgery, Faculty of Surgery, Red Cross War Memorial Children’s hospital, Cape Town, South Africa; 15 Department of Otolaryngology, Head and Neck Surgery, Mayo Clinic, Rochester, United States of America; 16 Department of Otolaryngology-Head and Neck Surgery, Division of Pediatric Otolaryngology, Stanford University, Stanford, United States of America; 17 Department of Aerodigestive and Airway Reconstruction Center, Stanford Children’s Health, Stanford, United States of America; 18 Division of Paediatric Otolaryngology, Head-Neck Surgery, Department of Surgery, Sidra Medicine and Research Center, Doha, Qatar

**Keywords:** paediatric, foreign body removal, sub-Saharan Africa, airway equipment, airway management

## Abstract

Sub-Saharan Africa (SSA), home to over 1 billion people, has only one paediatric otolaryngology fellowship program and nine fellowship-trained paediatric Otolaryngology Head and Neck Surgery (OHNS) specialists covering seven countries. Seven of these specialists estimated an average of 40+ patients per month are in need of critical surgical airway management in their respective countries and that 2–25 deaths per year (mainly paediatric) occur in their country from lack of access to foreign body removal equipment. Investing in paediatric airway infrastructure and capacity would largely benefit the health system in SSA, where the current lack of equipment alone leads to unmeasured morbidity and mortality. As a region of the world with the largest paediatric populations, sub-Saharan Africa’s need is all the more pressing given the disproportionately low number of medical specialists, institutions and resources. Collaborative approaches in procurement and maintenance of high-quality, cost-effective equipment are crucially desirable factors in both low- and middle-income countries (LMICs) and high-income countries (HICs). We, as the Global OHNS Initiative, urge for multi-stakeholder engagement and collaboration to forge lasting change.

Dr Fiona Kabagenyi recalls the following experience:

‘In 2015, I vividly remember a case when our Ear Nose and Throat [*ENT*]) team retrieved an aspirated molar tooth in a young boy. His family traveled 517 km, a 12+ h journey through 4 health centers, before reaching our team with the region’s only ENT surgeon at Mbale Regional Referral Hospital in rural Eastern Uganda. The center served a catchment area of 9 million people, the population of New York City. Roughly five aerodigestive foreign bodies [*FBs*], mostly in children, were referred weekly to this center. Given the long journey and barriers to seeking care–time, money, transportation – we likely only treated a fraction of the cases in the region.

During residency, I frequently looked through a bronchoscope with a broken prism and used 20-year-old forceps to retrieve FBs from the airway. I wore goggles to avoid the anesthetic gas entering my eyes when visualising the FB. There was also no flexible endoscope in the emergency theater.

To date, our pediatric ENT unit in Mulago National Referral Hospital receives many pediatric patients with complications due to long-standing airway FBs – including infections, FB migration, and airway stenosis. These patients require airway assessment with procedures like microlaryngoscopy and bronchoscopy; however, our hospital has yet to procure the equipment to perform this assessment. This is the true yet unfortunate reality – there are delays in presentation and a lack of life-saving airway equipment in tertiary facilities in low- and middle-income countries (LMICs), where 74% of the global population resides. If tertiary hospitals in LMICs all had functional, life-saving airway equipment coupled with expertise, the cumulative impact on the number of lives saved would be exponential.’ (Fiona Kabagenyi, MD, Pediatric Otolaryngologist, Makerere University and Mulago National Referral Hospital, Uganda)

## Paediatric airway infrastructure and capacity in sub-Saharan Africa

For 1 billion people, sub-Saharan Africa (SSA) has only one paediatric otolaryngology fellowship program and nine fellowship-trained paediatric Otolaryngology Head and Neck Surgery (OHNS) specialists covering seven countries. In an informal survey, seven of these specialists estimated an average of 40+ patients per month are in need of critical surgical airway management in their respective countries. Additionally, they estimated that roughly 2–25 deaths per year occur in their country from lack of access to foreign body (FB) removal equipment.

‘It’s like flying blind’, Dr. Samuel Okerosi at Machakos Level five Hospital in Kenya explains about navigating paediatric airway emergencies without proper endoscopic equipment. Some paediatric otolaryngologists in SSA have reported their airway endoscopic equipment to be up to 20 years old. Some centres only have access to one or two functional sets of equipment for airway FB removal, with limited ability to repair broken equipment. Three specialists reported having no access to flexible bronchoscopes. In addition to the lack of functional equipment, limited staffing and inefficient referral networks, respondents also reported limited availability to paediatric intensive care units (ICUs) and multidisciplinary subspecialities, such as cardiothoracic surgery, for open surgical removal of FBs refractory to endoscopic removal. Improved access to resources and basic equipment for diagnosis and retrieval of FBs is critical to reducing mortality and morbidity of the affected paediatric population in SSA.

Studies estimate the global mortality for paediatric inhaled FB emergencies to be 4% – 7%.^1^ Mechanical obstruction from FB is the primary source of fatal accidents in children under age 1 and a major cause of death in children aged 1–4 years.^2^ The burden of care for paediatric airway emergencies was highlighted in a Delphi methods study, which found paediatric FBs to have the second highest consensus agreement as a priority OHNS condition.^3^ A partner study determined that 5 of the 11 highest priority procedures in paediatric otolaryngology were related to airway obstruction.^4^ The Disease Control Priorities Third Edition emphasised the economic and a moral imperative that global partners invest in paediatric surgery as a vital component of reducing the burden of disease and improving the public health and economic fortunes of LMICs.^5^

## Essential airway equipment

**FIGURE 1:**
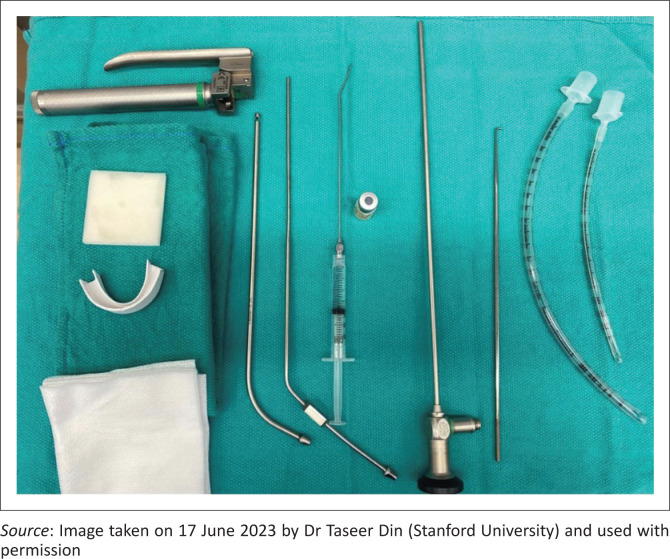
Left to Right: Phillips 1 laryngoscope, anti-fog (can be replaced with alcohol-based wipes), dental guard (replaceable with gauze), rigid suctions (pharyngeal, laryngeal), weight-based local anaesthesia with atomiser (atomiser can be replaced with a large-bore intravenous catheter), Hopkin’s telescope, right-angled probe, appropriate endotracheal tubes.

While challenges in strengthening health systems appear insurmountable, investing in basic essential equipment for diagnostic airway assessment is a tangible step (Figure 1).

**FIGURE 2:**
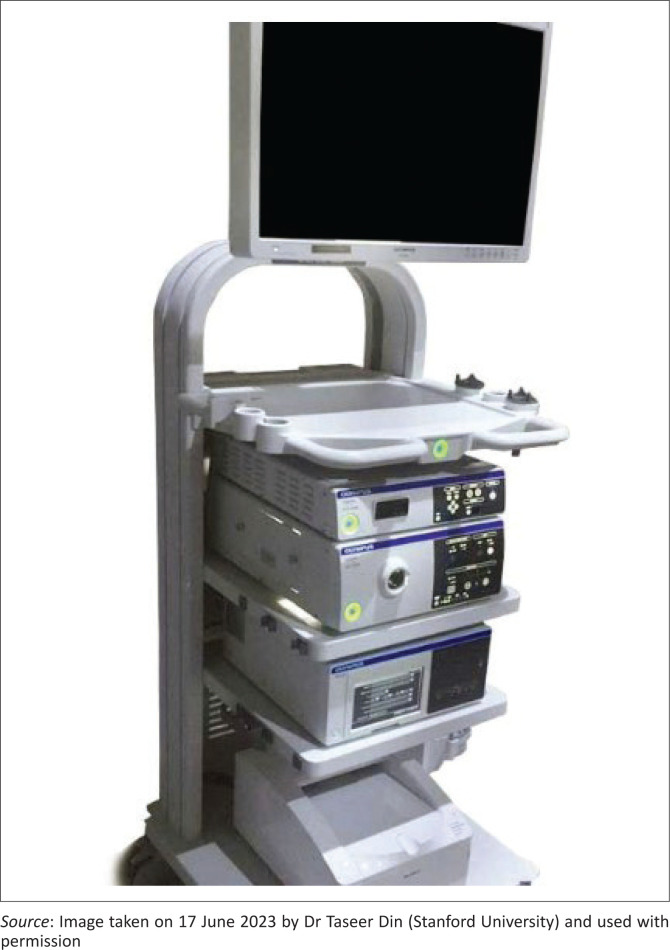
Endoscopy tower.

Having an ‘endoscopy tower’ (Figure 2) with a monitor, connecting camera-head and light source in tertiary institutions is ‘nice-to-have’, as it addresses needs across various subspecialties such as urology, general surgery and sinus and/or endoscopic ear surgery. Therefore, local concerting efforts can be made in procurement with investments utilised by multiple departments.

**FIGURE 3:**
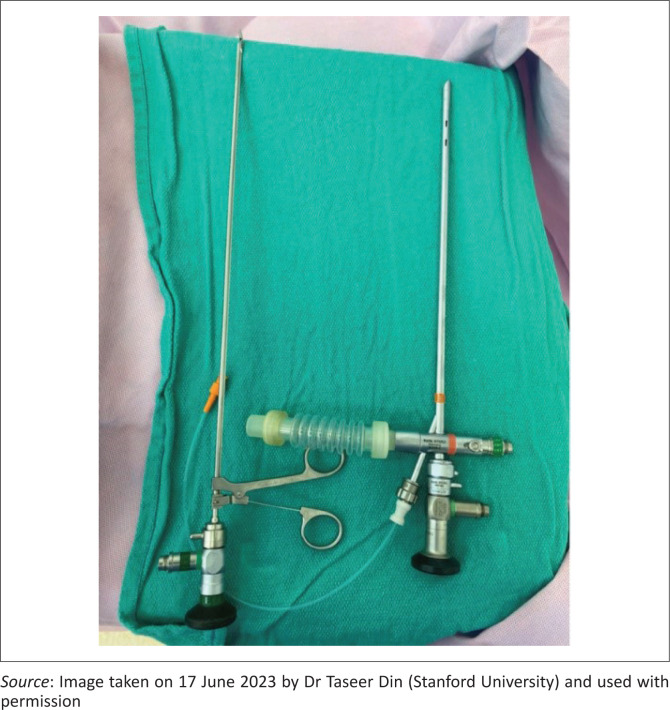
Left to Right: Optical grasping forceps; rigid bronchoscopy set up with an endoscope, ventilation port and suction catheter.

For interventional purposes, a rigid bronchoscopy set with optical forceps (Figure 3), a diagnostic laryngoscopy set up and a shared endoscopic tower can be effectively used to retrieve the vast majority of airway FBs.

Collaborative approaches in procurement and maintenance of high-quality equipment that are simple to set-up and cost-effective are crucially desirable factors in both LMICs and higher-income countries.

## Call to action

We call upon multiple stakeholders including surgical equipment companies, SSA health organisations, surgical societies, and non-profit and individual donors to engage in collaborative, context-appropriate and innovative solutions to alleviate these disparities. As healthcare practitioners, conscious about global surgical equity, we must (1) increase awareness of the urgent need to improve access to paediatric airway care, (2) promote essential airway equipment infrastructure, (3) invest in policies including global resource sharing and (4) collaborate with industry to develop distribution models in resource-limited health systems such as in SSA.

The passion and desire to improve outcomes in paediatric airway disease in SSA must be matched with the surgical infrastructure. As a region of the world with the largest paediatric populations, SSA has a need that is critical and pressing. We urge multi-stakeholder engagement and collaboration to forge lasting change.
